# Simulation and Experimental Studies on Grain Selection and Structure Design of the Spiral Selector for Casting Single Crystal Ni-Based Superalloy

**DOI:** 10.3390/ma10111236

**Published:** 2017-10-27

**Authors:** Hang Zhang, Qingyan Xu

**Affiliations:** 1 State Key Laboratory of Manufacturing System Engineering, School of Mechanical Engineering, Xi’an Jiao Tong University, Xi’an 710049, China; zhanghangmu@hotmail.com; 2 Key Laboratory for Advanced Materials Processing Technology, Ministry of Education, School of Materials Sciences and Engineering, Tsinghua University, Beijing 100084, China

**Keywords:** single crystal, spiral selector, directional solidification, structural parameter

## Abstract

Grain selection is an important process in single crystal turbine blades manufacturing. Selector structure is a control factor of grain selection, as well as directional solidification (DS). In this study, the grain selection and structure design of the spiral selector were investigated through experimentation and simulation. A heat transfer model and a 3D microstructure growth model were established based on the Cellular automaton-Finite difference (CA-FD) method for the grain selector. Consequently, the temperature field, the microstructure and the grain orientation distribution were simulated and further verified. The average error of the temperature result was less than 1.5%. The grain selection mechanisms were further analyzed and validated through simulations. The structural design specifications of the selector were suggested based on the two grain selection effects. The structural parameters of the spiral selector, namely, the spiral tunnel diameter (*d*_w_), the spiral pitch (*h*_b_) and the spiral diameter (*h*_s_), were studied and the design criteria of these parameters were proposed. The experimental and simulation results demonstrated that the improved selector could accurately and efficiently produce a single crystal structure.

## 1. Introduction

Single crystal blades have been widely utilized in aviation and energy, due to the corresponding superior high-temperature performance. The blades serve in extremely high-temperature and high-stress environments in turbine engines. Crystal defects in single crystal blades—such as stray grains and double crystals—can highly decrease the pass rate of the products during directional solidification (DS) [1,2]. Certain defects appear during the initial stage of single crystal formation, which has attracted an increasing attention from scholars.

Single crystal structures are mainly obtained during directional solidification by way of two methods—grain selection and seed crystal methods. In the seed crystal method, a specific orientation seed is placed at the bottom of the casting shell, where the seed grows through the deposition of metal atoms during DS, consequently producing a single crystal structure. In this method, a fusion zone exists between the melt and the seed crystal. The grain growth region transitions from unsteady self-organization to steady-state growth, as well as the solidification behavior are affected by additional factors. Therefore, this process is hard to control [3,4,5]. At present, the grain selection method is widely utilized [6,7,8,9,10]. Spiral grain selection is a common grain selection method, which has advantages such as simple processing, short production cycle and high operability [11]. A spiral selector has a complex spatial structure, which creates a complex visibility relationship among the selector, the furnace heating and the cooling wall. Consequently, both heat transfer and microstructure growth are complex and hard to control.

The key components of a spiral selector include the starter block and the spiral part. The starter block mainly completes the grain chilling nucleation and competitive growth at a certain height, to ensure that a certain number of grains grow into the spiral part. The main role of the spiral part is to remove the majority of grains, according to the geometric structural constraints, as well as to only select crystals with low orientation deflection angles (the angle of the [001] preferential grain orientation that deviates from the DS withdrawal direction), which are lower than 15° in the industry. Trial and error are mainly used in the current design of spiral selectors. Research on crystal selection and selector structure designs has been conducted by scholars with analytical, experimental and simulation methods. Carter et al. [8] conducted a temperature experiment and analyzed the temperature gradient and cooling rate effects on the initial grain competitive growth. Epishin and Nolze [12] studied the regularity of grain orientation with the height of the solidification through the electron backscatter diffraction (EBSD) cross section observation of a spiral selector. D’Souza et al. [13] studied the isothermal curvature effect on the grain orientation of the selected crystal. Zhou et al. [14] proposed a grain competition model by studying the competitive growth of bi-grain orientations and improving the grain competition model that was proposed by Walton and Chalmers [15]. Jiang et al. [16] studied the grain selection process of Ni_3_Al alloys and explained the relationship between the grain orientation and the phase. Meng et al. [17] studied the changes in grain density with the ingot height. Liu et al. [18] explained the competitive growth mechanism of dendrites from the orientation distribution characteristics of primary and secondary dendrites.

As for the selector structure design, Carter et al. [8] studied the thermal equilibrium process and grain growth in a spiral selector with experimental and simulation techniques, proposing an optimized squared-helix selector. Dai et al. [10,19] designed various spiral selector structures and studied the effects of different structural parameters on crystal selection. Seo et al. [20] studied a hard-turning grain selector and presented an EBSD grain orientation. Zhang et al. [21] designed spiral selectors with different spiral angles, and the results show that the crystal selector with a smaller spiral angle could effectively reduce the deviation of preferred orientation. Wang et al. [22] adopted ProCAST&CAFE model to simulate grain selection behavior with different spiral geometries. They proposed that the coupling effect of the heat flow direction, the preferred growth direction and the geometrical restriction of the spiral wall makes a contribution to the crystal selection in the spiral passage. There are other simulation methods used to model competitive grain growth, such as envelope method and phase-field model [23], which still attract researchers’ attentions.

At present, most of these studies still involved qualitative research. The selection mechanism and law of the spiral selector remain to be studied. The design of grain selectors also lacked corresponding theoretical guidance. In previous works, the grain selection simulation was executed and the spiral grain selection mechanism was proposed [6,24]. In this work, the heat radiation model and the grain growth model concerning solute equilibrium were added. The relationship between the grain selection mechanism and the DS process were analyzed further, based on a simulation tool. This led to a proper guide for the selector structure design. In corresponding experiments, a variety of spiral selectors were directly fabricated based on the 3D printing method and DS pouring experiments were conducted. Finally, the selector structural design parameters effects on crystal selection were investigated for the design criteria to be proposed.

## 2. Models and Methods

### 2.1. Physical and Mathematical Model

#### 2.1.1. Energy Conservation Models

Energy transfer during DS is significantly complicated. The heat transfer method includes: (1) the heat radiation effect of the upper heater on the shell; (2) the heat radiation from the shell to the cooling zone; (3) the heat flow out from the chill; (4) the heat conduction between the shell and the casting; (5) the internal heat conduction of the casting; and (6) the heat radiation between the shells. The main heat transfer methods could be divided into two categories—heat radiation and heat conduction.

In terms of the latent heat release of a metal, an energy conservation model can be described by a uniform Equation [24] as presented below:

(1) $$c\rho \frac{\partial T}{\partial t}=\left[\frac{\partial }{\partial x}\left(\lambda \frac{\partial T}{\partial x}\right)+\frac{\partial }{\partial y}\left(\lambda \frac{\partial T}{\partial y}\right)+\frac{\partial }{\partial z}\left(\lambda \frac{\partial T}{\partial z}\right)\right]+Q_{M}$$

where, *Q*_M_ is the latent heat source. When the material is a shell, core or other non-latent heat release material, *Q*_M_ is 0; when the material is a metal and a phase change occurs, $Q_{M}=\rho L\cdot \partial f_{S}/\partial t$.

The heat radiation between two-body (α and β) surfaces is controlled by the Stefan-Boltzmann law. This equation can be expressed as follows:

(2) $$Q_{\alpha -\beta }=\sigma \left(T_{\alpha }^{4}-T_{\beta }^{4}\right)/\left(\frac{1-\varepsilon_{\alpha }}{\varepsilon_{\alpha }}\cdot \frac{1}{A_{\alpha }}+\frac{1}{\varphi_{\alpha -\beta }}\cdot \frac{1}{A_{\alpha }}+\frac{1-\varepsilon_{\beta }}{\varepsilon_{\beta }}\cdot \frac{1}{A_{\beta }}\right)$$

where, *φ*_α−β_ is calculated by Equation (3). A mathematical method to solve the radiation angle during DS involves a high amount of calculations and high memory footprint. The Monte Carlo method [25] is utilized to discretize the visible surface of the radiation, to calculate the angle coefficient between the finite numbers of visible surfaces, as well as to obtain the radiation heat transfer of each surface element:

(3) $$\varphi_{\alpha -\beta }=\frac{C_{V}}{A_{\alpha }}\int_{A_{\beta }}\int_{A_{\alpha }}\frac{\cos\theta_{\alpha }\cos\theta_{\beta }}{\pi R^{2}}dA_{\alpha }dA_{\beta }$$

#### 2.1.2. Grain Nucleation and Growth

At the initiation of DS, the bottom of the superalloy melt is chilled by the water-cooled chill, whereas the high-sized atomic clusters are attached to the chill surface to nucleate and grow. This process can be described by the instantaneous nucleation model as follows:

(4) $$\frac{dN}{dt}=K_{1}(n_{0}-n_{t})\exp(-\frac{K_{2}}{\Delta T^{2}T})$$

In this model, the nucleation rate of the melt is related to the nucleation density and the undercooling degree. When a high-temperature melt is reduced to a certain value, a high amount of nuclei form.

The grain growth is simultaneously affected by the heat transfer and solute transport. The solute transport is controlled by the following equation:

(5) $$\frac{\partial C_{i}}{\partial t}=D_{i}\left(\frac{\partial^{2}C_{i}}{\partial x^{2}}+\frac{\partial^{2}C_{i}}{\partial y^{2}}+\frac{\partial^{2}C_{i}}{\partial z^{2}}\right)i=L,\;S$$

As the DS proceeds, the grain growth velocity *v*_n_ can be calculated in terms of the solute conservation at the solid/liquid (S/L) interface, as follows:

(6) $$v_{n}C_{L}^{*}\left(1-k_{e}\right)=D_{L}\left(\frac{\partial C_{L}}{\partial x}+\frac{\partial C_{L}}{\partial y}+\frac{\partial C_{L}}{\partial z}\right)-D_{S}\left(\frac{\partial C_{S}}{\partial x}+\frac{\partial C_{S}}{\partial y}+\frac{\partial C_{S}}{\partial z}\right)$$

The solute conservation equation and the S/L interface solute transport are calculated according to Equations (5) and (6). At the S/L interface, the solute distribution meets the leverage criterion:

(7) $$C_{S}^{*}=k_{e}C_{L}^{*}$$

where, the liquid phase concentration at the S/L interface is calculated by the following equation [26]:

(8) $$C_{L}^{*}=C_{0}+\frac{1}{m_{L}}\cdot \left[T^{*}-T_{Liq}+\Gamma \kappa f\left(\theta_{i}\right)\right]$$

where, Γ is the Gibbs-Thomson coefficient, $\Gamma =\gamma_{V}/\Delta S$; $\gamma_{V}$ is the interface energy per unit volume; ΔS is the melting entropy; κ is the interface curvature; and $f\left(\theta_{i}\right)$ is the interface anisotropy function. κ and $f\left(\theta_{i}\right)$ can be calculated by the following equations [26,27]:

(9) $$\kappa =\frac{1}{a_{m}}\left[1-\frac{2}{125}\sum_{i=1}^{125}f_{s}(i)\right]$$

(10) $$f\left(\theta_{i}\right)=\prod_{i=l,m,n}\left[1+\gamma \cos(K_{i}\theta_{i})\right]$$

where, $f_{s}(i)$ is the solid phase rate of the adjacent *i*-cell; *a*_m_ is the step size of the micro cell; *γ* is the anisotropy intensity, *K_i_* is the anisotropy modulus and *θ_i_* is the interface anisotropy angle.

### 2.2. Materials and Methods

#### 2.2.1. Temperature Measurement Experiment

**Figure 1:**
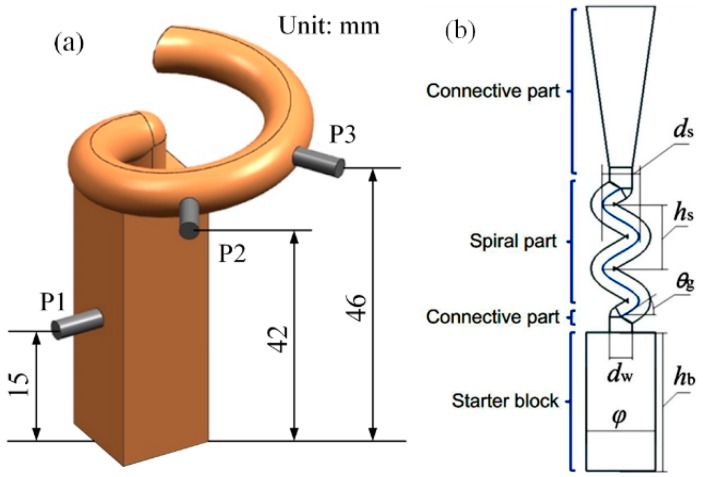
Temperature measurement experiment: (**a**) arrangement of measuring points and (**b**) structure of typical spiral selector.

A temperature measuring experiment was conducted to verify the heat transfer model. A common spiral grain selector could be divided into three components: the starter block, the spiral part and the transition part. The starter block and spiral part are the key components in Figure 1b. Therefore, three key measurement points were set in the experiment selector in Figure 1a. One point was set in the center of starter block and the other two were set in the spiral tunnel.

The standard thermocouple WRe5/26 of 0.5 mm in diameter and with 0.1% deviation under 673–1873 K (400–1600 °C) was used. The temperature sampling device was an IMC 32 channel data acquisition instrument, for which, the sampling period was 200 ms and the sampling time was 4 h.

The process of the DS experiment was as follows: Heating–Melting–Pouring–Standing–Withdrawal–Cooling. The pouring temperature was 1773 K (1500 °C), the cooling water temperature was 313 K (40 °C), the drawing speed was 5 mm/min and the shell was 6 mm in thickness. The material was a DD6 single crystal superalloy [28].

#### 2.2.2. Microstructure Experiment

The main structural parameters of the spiral part included the spiral pitch *h*_s_, the spiral diameter *d*_s_, spiral angle *θ* and the spiral channel diameter *d*_w_, as presented in Figure 1b. Among these parameters, the *h*_s_, *d*_s_ and *θ* have the following relationship [10]:

(11) $$h_{s}=\pi \cdot d_{s}\cdot \tan\theta$$

To precisely control the spiral part with different structural parameters, the 3D printing technology was utilized to precisely shape the spiral selectors. Spiral selectors with various parameters were designed by the Unigraphics NX 6.0 software (V6.0, Siemens PLM Software, Plano, TX, USA). The parameters are listed in Table 1. The chill radius *R* was 100 mm and the eccentric radius *r* was 72.5 mm.

**Figure 2:**
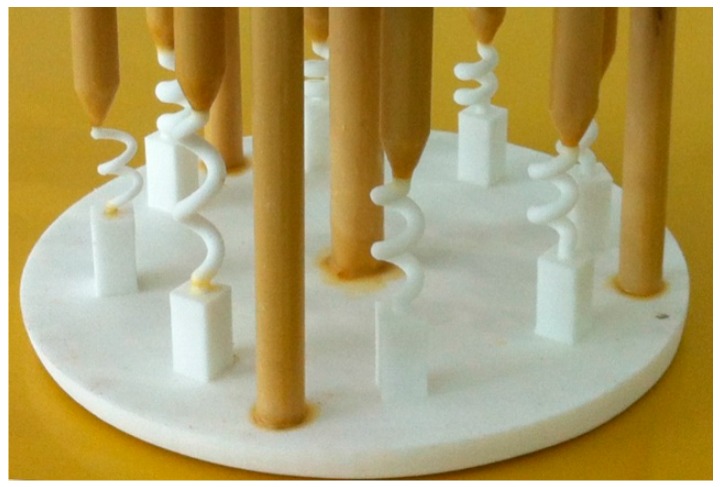
White wax mold fabricated by 3D printing.

Subsequent to conversion into the STL file format, the casting model was directly printed using ProJet CPX3000 Plus equipment—the white wax mold from this 3D printing is presented in Figure 2. Consequently, the selectors were solidified alongside other processes.

Following solidification, the spiral part was cut and cross sections were created every 90° along the spiral. These sections were named Sp1-Sp9. The metallographic etchant was composed of HCl (37%, 5 mL) + HF (48%, 2 mL) + Cu_2_SO_4_.5H_2_O (99.9%, 2 g) + H_2_O (23.5 mL). Scanning electron microscope (SEM) and EBSD observations were performed with a JSM-6310F device.

#### 2.2.3. Simulation Method

The DS processes of the single crystal selector were simulated based on the models built, coupled with the CA-FD method. The material and process parameters in these simulations were similar to the corresponding experiments. Visual C++ was used for the programming. The calculation platform was an Intel P4 3.6 GHz host with a Windows Server 2003 operating system. Table 2 presents the physical properties that were used in the simulation.

## 3. Results and Discussion

### 3.1. Temperature Comparison

The simulating results of temperature variation were compared to the thermal measurement results, as presented in Figure 1. The continuous decreasing tren *d*_s_ of temperature in each point were in agreement with the temperature variation properties during the DS process. The complex structure of the spiral selector led to the visual relationship becoming complicated, whereas the complicated heat radiation changed the temperature curve of P2, which was supposed to be “parallel” to the others. The measured temperature of P1 at the initial solidification was apparently lower compared to the P2 and P3 measured temperatures, whereas for the later solidification, the corresponding temperatures of the three points from the simulation were basically identical. This result occurred because the high-temperature metal melt was chilled by the water-cooled chill at the bottom of the selector, which decreased the initial temperature of P1. The temperature curves along the 3 points from the simulation and experiment fitted well and the corresponding average errors were below 1.5%.

**Figure 3:**
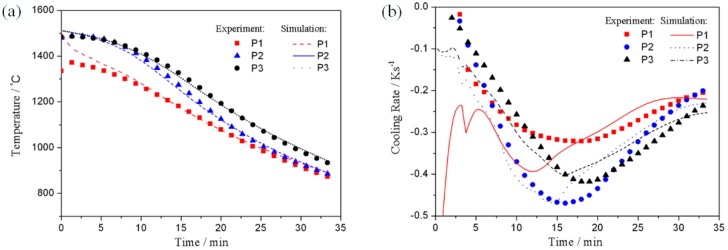
Comparison of temperature and cooling rate curves from experiment and simulation: (**a**) temperature change with time and (**b**) cooling rate curve.

Figure 3b presents that the absolute values of the three points’ cooling rates first increased and consequently decreased. As the DS proceeded, the 3 points were gradually drawn into the cooling zone and following this, the cooling rate increased. On the other hand, the temperature differences between the measuring points and the heat dissipating sources decreased as the temperature of each point decreased. Consequently, the cooling rates decreased. The simulation results demonstrated that the cooling rate was basically identical to the cooling rates of the temperature measurement results; it was noticeable that the simulation fluctuation of P1 at the initial stage was also caused by the chilling effect.

### 3.2. Microstructure Evolution

#### 3.2.1. Grain Evolution in Starter Block

**Figure 4:**
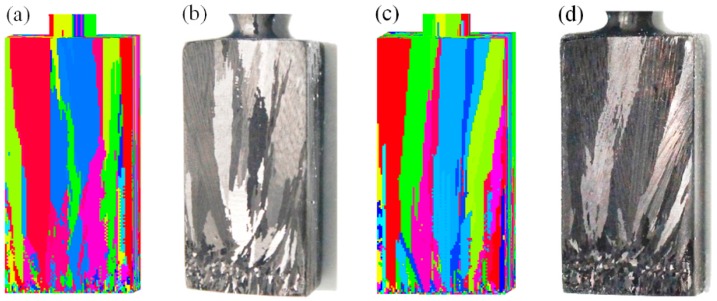
Comparison of microstructures in real starter block of spiral selector from experiment and simulation: (**a**) simulation result (front side); (**b**) experimental result (front side); (**c**) simulation result (reverse side) and (**d**) experimental result (reverse side); starter block dimensions: 10 mm × 10 mm × 30 mm.

The microstructure evolution during DS could be further simulated, based on accurate heat transfer calculations. Figure 4 presents a comparison of the grain growth in a real starter block by simulations and experiments. At the beginning, a high amount of low-sized grains existed at the bottom region, whereas the sizes gradually increased as the solidification height increased. When the height exceeded a certain value, the grain sizes tended to remain unchanged. In this figure, the grain morphology and distribution matched the experimental results grain morphology and distribution quite well. The numerical models could successfully reproduce the grain growth and evaluation of the starter block during DS.

**Figure 5:**
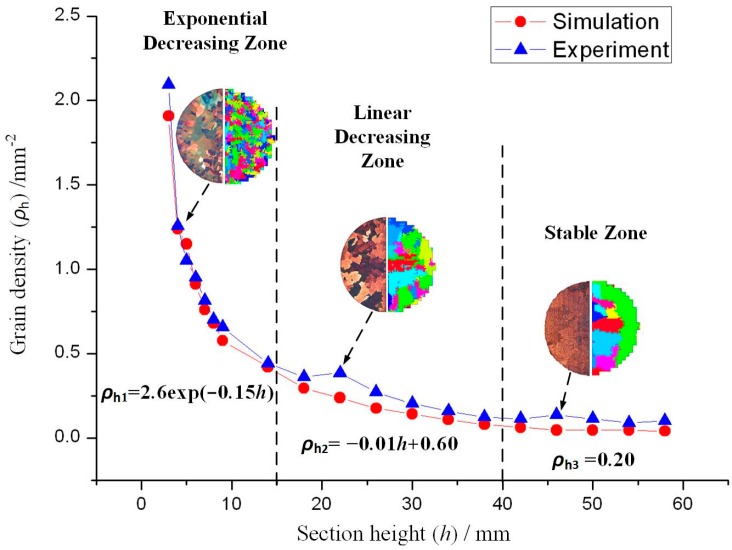
Comparison of grain density changes with section height during experiment and simulation.

In the previous study [24], the grain density curve of the starter block could be divided into three sections according to the grain density distribution: the exponentially decreasing zone (*ρ*_h_ = 2.61exp(−0.15*h*)), the linearly decreasing zone (*ρ*_h_ = −0.01*h* + 0.60) and the stable zone(*ρ*_h_ = 0.20), as presented in Figure 5. This had significant meaning during the height design of the starter block, which was discussed further.

#### 3.2.2. Grain Evolution in Spiral Part

**Figure 6:**
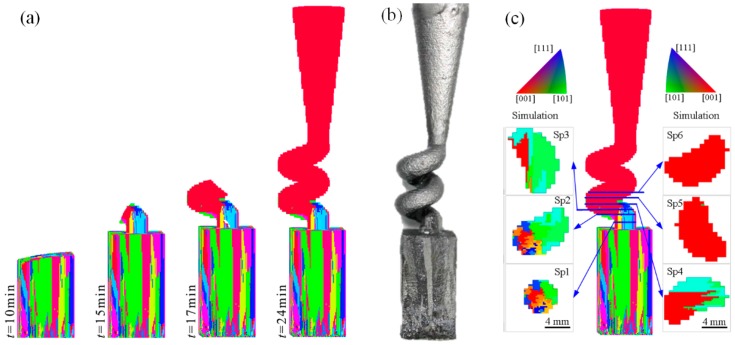
Comparison of grain growth in spiral part during experiment and simulation: (**a**) microstructure evolution simulation; (**b**) experimental result and (**c**) comparison of microstructure morphologies in different sections; structure of spiral part: *d*_s_ = 6 mm and *h*_s_ = 10.9 mm, *θ* = 30° and *d*_w_ = 5 mm.

The grain growth and evolution in the spiral part were simulated, as presented in Figure 6. Following the starter block solidification, the grains entered the spiral part. In this case, the single crystal was successfully selected in 17 min, whereas the longitudinal growth height (the distance between the single crystal appearance height and the start height of the spiral part) did not exceed 10.9 mm in the spiral part. The S/L interface along the spiral part was apparently inclined, which meant that the unidirectional heat flow was completely destroyed. This result occurred because the heat-transfer efficiency through the metal was higher compared to the heat-transfer efficiency through the shell. Most heat flowed along the spiral tunnel, resulting in the inclination of the isothermal surface and the S/L interface. Figure 6c presents the grain distributions in different sections of the spiral part. According to these simulation results, the number of grains in Sp1 was higher, whereas the grain size was lower. In Sp2, the grains on one side of the section increased in size and occupied the local region. As this process continued, the number of grains in the other areas of this section gradually decreased. When the grains reached Sp4, only one grain was selected as the final single crystal.

**Figure 7:**
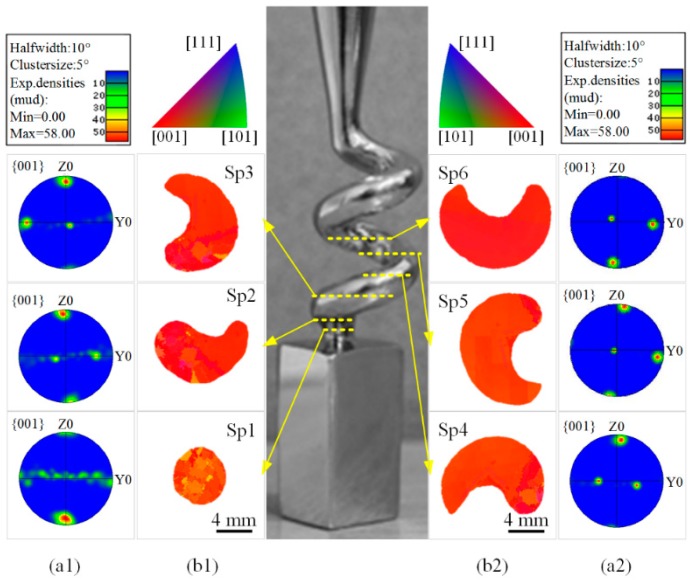
EBSD orientation imaging microscopy (OIM) of different cross sections in spiral part (**a1**) and (**a2**): pole figures (PF); (**b1**) and (**b2**): inverse pole figures (IPF); structure of spiral part: *d*_s_ = 12 mm, *h*_s_ = 14 mm, *θ* = 20° and *d*_w_ = 5 mm.

The grain competition growth in the spiral part was further studied with an EBSD experiment, as presented in Figure 7. When the grains grew into Sp1, all the [001] orientations were close to the z axis, whereas the [100] and [010] orientations were evenly distributed along the *x*O*y* surface, as presented in Figure 7a1. The area that was occupied by the highest-sized grain was only 26.1% (Table 2), that is, at least 5 grains grew into the spiral section. When the grains entered Sp2, the [001] orientations were still close to z axis, while the points for the [100] and [010] orientations existing in the pole figure, which were originally dispersed along the *x*O*y* surface, began to focus. The maximum grain area was 40.3%. These meant that certain grains were eliminated. In sections Sp3−Sp6, the point cloud along the [001], [100] and [010] orientations converged to a low-sized local zone in the pole figure, whereas the area that was occupied by the highest-sized grain changed from 76.1% to 100%, while the angle between the [001] orientation of the highest-sized grain and the z axis was 9.29° on average. The highest-sized grain in Sp4−Sp6 was already the final single crystal, whereas the deviations in the orientation angles were different in these sections. These differences were mainly caused by the inclination of the cross section, when the samples were cut and ground. In contrast, these differences were small, proving that the crystal selection process was successful.

As shown in Figure 7b1,b2, the grain orientations in the inverse pole figures (IPF) tended to be centralized in the [001] direction—the grain morphologies and distributions of the EBSD results matched the simulation results in Figure 6c. In particular, the selection behavior in both the simulation and the experiment demonstrated one grain growing on one side of the section, quickly increasing in size and finally occupying the entire section.

### 3.3. Simulation Analysis and Validation of Selection Mechanism

There are certain studies regarding the grain selection mechanisms in the spiral part. Gao et al. [29] proposed that the main mechanism of grain selection in the spiral part was geometrical blocking. Dai et al. [19] emphasized that the efficiency of the spiral selector was significantly dependent on the corresponding geometry, whereas the spiral became quite efficient with (a) lower pitch length; (b) lower take-off angle; (c) lower wax wire diameter and (d) higher spiral rotation diameter at the same pitch length. Zhang et al. [6] reported two grain selection effects, promoting the effect in the horizontal direction (Effect 1) and the primary dendrite growth restraint effect in the vertical direction (Effect 2) of the spiral part. In this study, to further present the relationship between the grain selection effects and the selector structure, proving a guide for the design of different spiral selectors, the simulation was utilized for further analysis and verification.

**Figure 8:**
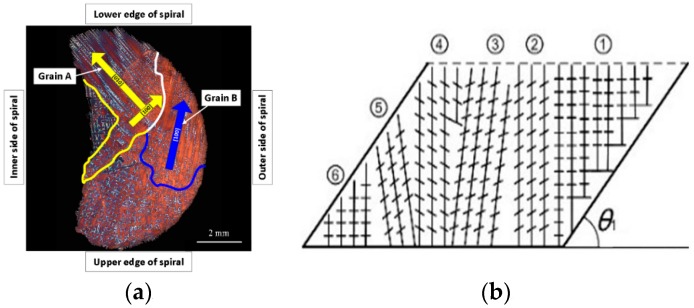
Schematic of grain selection mechanism of spiral part [6]. (**a**) secondary dendrite growth promoting effect in horizontal direction (named Effect 1) and (**b**) primary dendrite growth restraints effect in vertical direction (named Effect 2).

During a grain selection process, most grains were strained and eliminated in the spiral part. The grains near the lower edge of the cross section through the spiral part expanded and grew in horizontal directions, as presented in Figure 8a. The secondary dendrites A and B grew along the [100] preferential direction. In contrast, the secondary dendrites of grain B were blocked by the outer side wall of the spiral part and the trunks of grain A. Consequently, grain B had no growth advantage and was eliminated. Therefore, the grains near the lower edge and the inner side of the spiral tunnel had a better positional advantage during the competitive growth. The geometric constraints of the spiral section enhanced the selection effect in the horizontal section by promoting the competitive growth of secondary dendrites in the [100] and [010] directions.

The spiral angle *θ* of the spiral selector provided external geometric constraints in the vertical direction to restrain certain grains. Figure 8b illustrates the restraint and elimination of grains by the inclined tunnel wall. The grains ⑤ and ⑥ were eliminated with the spiral angle *θ*_1_. When the spiral angle decreased, a higher number of grains (including ②−⑥) might be blocked and eliminated. Therefore, the spiral selector exhibited a direct geometric constraint on the growth of primary dendrites from the grains in the vertical direction.

**Figure 9:**
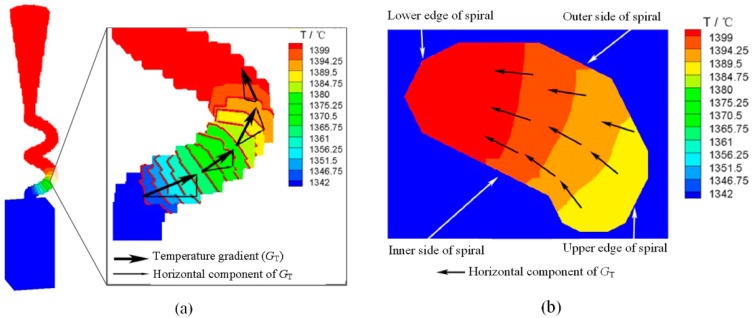
Simulation result of spiral temperature field distribution: (**a**) local temperature distribution of selector and (**b**) temperature gradient horizontal component in cross section.

**Simulation analysis of Effect 1:** The simulation models were built to demonstrate the further relationship between the grain selection effect and the process. As for Effect 1, mainly two external factors existed: (1) a horizontal temperature gradient condition that promoted dendrite growth in the [100] and [010] directions; and (2) the complex geometric structure of the cross section. For external factor (1), the simulation result demonstrated a horizontal component to the temperature gradient along the spiral cross section—as presented in Figure 9b—mainly because the thermal conductivity of the alloy was quite higher compared to the shell (approximately 17 times higher, Table 2). Consequently, the heat transfer along the spiral channel was the main heat transfer path. Following, a horizontal component for the temperature gradient existed. Subsequently, if the spiral angle (*θ*) was lower, the horizontal component of temperature would be higher, which led to a significant rapid growth of the secondary dendrite, which meant that the selection effect was enhanced. As for external factor (2), the width and curvature of the cross section affected the grain selection effect also. As an example, if the tunnel diameter (*d*_w_) was higher, the cross section of the spiral tunnel would be wider and the curvature of the cross section would be lower. Consequently, the constraint effect by the spiral tunnel was weakened.

**Figure 10:**
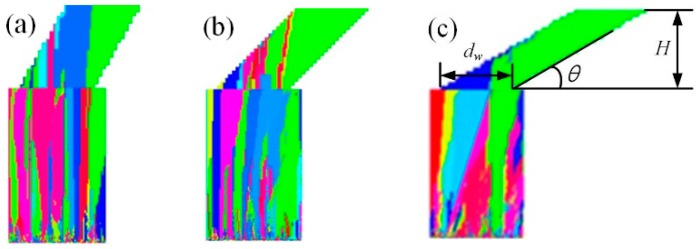
Simulation analysis of grain selection effect for different spiral angles (*H* = 14 mm and *d*_w_ = 12 mm): (**a**) *θ* = 60° and *h* > 14 mm, (**b**) *θ* = 45° and *h* = 14 mm, and (**c**) *θ* = 30° and *h* = 10 mm; *h* is appearance height of single crystal.

**Simulation analysis of Effect 2:** In addition, inclined selector models were constructed to verify Effect 2 and the simulation results are presented in Figure 10. In this figure, the inclined tunnel height *H* was 14 mm and the tunnel width *d*_w_ was 12 mm. When the inclined angle was 60°, the single crystal selection process did not finish at the solidification height *H*; when the angle was 45°, the solidification height *H* was the critical height for a single crystal selection; when the angle was 30° at the solidification height of *h* = 10 mm, a single crystal structure was successfully selected prior to the tunnel height *H* was reached. It could still be proved that the geometric shape of the spiral tunnel did not change the growth directions of these columnar grains [29]. The grains just were blocked by the tunnel wall, as presented in Figure 10. Apparently, the lower the *θ* was, the stronger the vertical constraint effect would be.

Moreover, the geometrical constraint by the spiral angle was related to the tunnel diameter *d*_w_. In Figure 10, when the design height *H* was higher than *d*_w_·tan(*θ*), the geometric constraint effect of the spiral angle completely played out and only grains that were close to the right edge of the spiral were selected. Therefore, the spiral pitch *h*_s_ must match Equation (12). If the grains grew to the height *h*_s_, the effect of Effect 2 completely played out.

*h*_s_ ≥ *d*_w_·tan(*θ*) (12)

**Figure 11:**
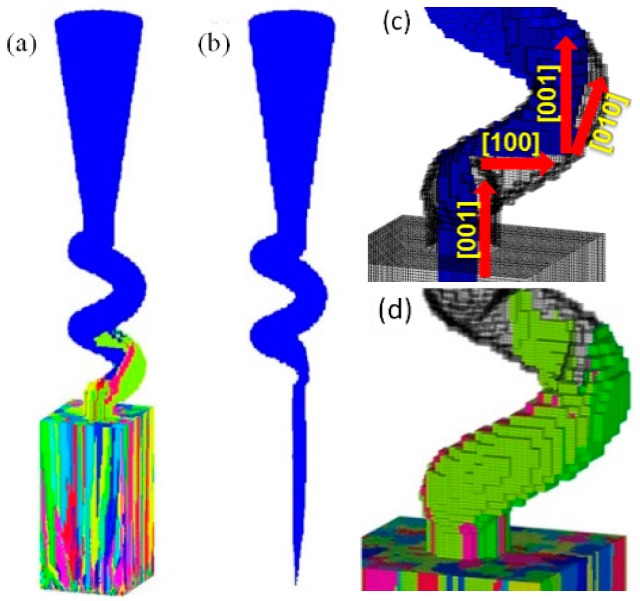
Simulation analysis of general spiral grain selection process: (**a**) microstructure in selector; (**b**) single crystal growth during entire selection; (**c**) single crystal growth directions in local spiral part; and (**d**) microstructures of eliminated grains.

The actual grain selection occurred in a 3D spiral tunnel, based on a combination of the aforementioned two effects. Figure 11 presents the grain competition growth and elimination process in the 3D selector simulation. In Figure 11c, the grain with a positional and orientation advantage grew into the spiral part, which further exhibited horizontal growth in the [100] direction and lateral growth in the [010] direction, finally dominating the competitive grain growth. Figure 11d presents that the other grains that entered the spiral part also grew at a certain height, whereas the growths were blocked by the spiral tunnel wall and the dominating grain, being eventually eliminated.

## 4. Selector Structure Design

### 4.1. Effects of Structural Parameters on Grain Selection

There are several structural parameters (*h*_b_, *φ*, *h*_s_, *d*_s_, *θ* and *d*_w_) of a spiral selector. The relationships between the selector structural parameters and the grain selection were investigated based on experiments and simulation.

#### 4.1.1. Effects of *h*_b_ and *φ*

According to Section 3.2, the *h*_b_ mainly affected grain density during the selection process. In terms of grain density variations, the number of grains that entered the spiral part was controlled, while a higher *h*_b_ value might have consumed additional metal and time. Commonly, if *d*_w_ = 5 mm, which should permit 5–6 grains to enter the spiral part, *h*_b_ should be located in the linearly decreasing zone with the fitting equation of *y* = −0.01*h* + 0.60. Consequently, the design range of *h*_b_ was suggested to be from 30 mm to 35 mm.

*φ* is the diameter of the cylindrical starter block. During the initial stage of DS, the temperature gradient was high and the isothermal surface was basically retained horizontal. Therefore, the cross-section size of the starter block barely affected the crystal selection. Cylinders and quadruple prisms were both commonly used in the industry. The design required that *φ > d*_w_ and the general range for *φ* was suggested from 10 mm to 15 mm.

#### 4.1.2. Effects of *d*_w_

**Figure 12:**
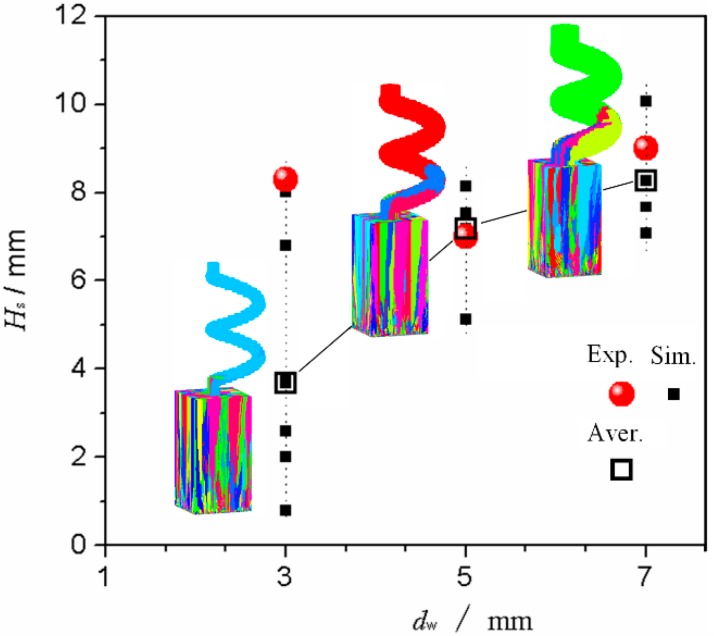
Comparison of *H*_s_ in selectors with different *d*_w_, during experiment and simulation (5 trials) with *h*_s_ = 14 mm and *θ* = 30°.

The spiral tunnel *d*_w_ limited the number of grains that entered the spiral part. Experiments and simulation trials were conducted to further determine the effect of *d*_w_ on grain selection. When a certain section was completely filled with a single crystal structure, the distance between this section and the bottom of the spiral part could be defined as the single crystal selected height (*H*_s_). Figure 12 presents a comparison of *H*_s_ in selectors with different *d*_w_. When the *d*_w_ increased, the average value of *H*_s_ increased and the grain selection effect was weakened, mainly because the number of grains that entered the spiral part was lower and the crystal selection process was completed significantly quickly when the *d*_w_ was thinner. In addition, the crystal selection range was wide when the *d*_w_ was low. Consequently, the apparent position of *H*_s_ was unstable. This phenomenon occurred because the number of grains that entered the narrow tunnel was low and the grain selection randomness was enhanced. Contrarily, the effect of the grain selection’s randomness was weakened and the appearance height of single crystal (*H*_s_) appeared more concentrated when the *d*_w_ was high. Additionally, all the results for *H*_s_ were lower than 14 mm along a spiral pitch under these study conditions, which indicated that the use of a 1-turn spiral structure was sufficient for the single crystal selection.

The *d*_w_ could be set to a range from 5 mm to 6 mm, even though the *d*_w_ significantly affected the crystal selection process, including the structural strength of the selector and the choice of selection efficiency. In addition, a 1-turn spiral structure was sufficient and proposed.

#### 4.1.3. Effects of *h*_s_

**Figure 13:**
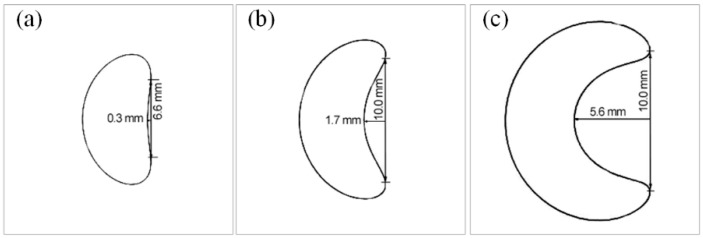
Cross-section shapes of spiral part with different *h*_s_ (*d*_s_ = 12 mm and *d*_w_ = 5 mm): (**a**) *h*_s_ = 8.7 mm, *θ* = 13.0 and BD = 0.09; (**b**) *h*_s_ = 14.0 mm, *θ* = 20.2° and BD = 0.3; and (**c**) *h*_s_ = 20.1 mm, *θ* = 28.1° and BD = 1.12.

According to Equation (11), if the *d*_s_ is constant, the *h*_s_ is proportional to tan*θ*. According to the external factors of Effect 1, the geometry of the horizontal section crucially affected the grain selection process: the more complex the cross section was, the stronger the grain selection effect would become. Figure 13 presents a comparison of the cross-section shapes of a spiral selector with different *h*_s_. All cross sections displayed an approximate “crescent shape”. To quantitatively express the complexities of these cross sections, a characteristic parameter, the bending degree (BD), was proposed to describe the bending structure of the cross section, which is defined as follows:

BD = 2*H_a_*/*L_c_* (13)

where, *H_a_* is the height between the inner arc and the corresponding chord, where *L*_c_ is the chord length. Therefore, the BD of the cross section was higher and the Effect 1 would be stronger.

**Figure 14:**
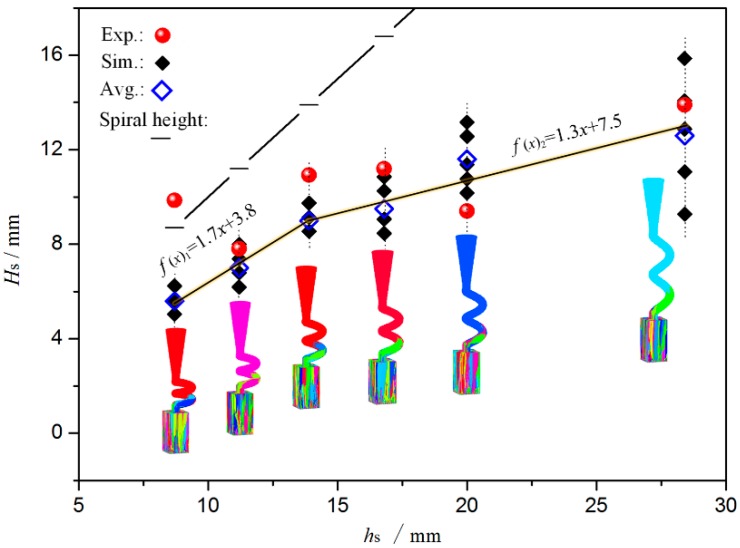
Comparison of *H*_s_ in selectors with different *h*_s_ during experiment and simulation (5 trials) with *d*_s_ = 12 mm and *d*_w_ = 5 mm.

In Figure 13, the BD increased from 0.09 to 1.12 and the *θ* increased from 13.0° to 28.0°, when the *h*_s_ increased from 8.7 mm to 20.1 mm. These changes affected the grain selection from two perspectives: (1) if the BD increased, the selection effect was enhanced (Effect 1); and (2) if the *θ* increased, the selection effect was weakened (Effect 2). Therefore, the comprehensive effects of selection must be determined according to the actual grain selection results and thus the experimental results for the ES1 group were analyzed through EBSD to determine the *H*_s_ values of the different selectors and a series of simulation trials were conducted, as presented in Figure 14. Increasing the *h*_s_ produced two results: (1) The average value of *H*_s_ slowly increased, which indicated that the coupling result of the two opposite effects weakened the grain selection. Two linear fitting curves were created, the $f^{\prime }{\left(x\right)}_{1}=1.7$ and $f^{\prime }{\left(x\right)}_{2}=1.3$. Consequently, the increasing slopes were different and the curve turning point was located at *h*_s_ = 14 mm; and (2) The range of *H*_s_ gradual widening. Consequently, the randomness of the grain selection process was amplified. Here the randomness of the grain selection process included the randomness in the orientation of single crystals and the randomness in the selected height.

In addition, the experimental results were higher than the simulation results, especially when *h*_s_ = 8.7 mm and the experimental *H*_s_ exceeded 8.7 mm. During actual DS, the orientations of dendrites might be affected by deflections in both the temperature gradient and solute convection, which might increase the *H*_s_. This factor was not considered in the current simulations. Therefore, the experimental *H*_s_ exceeded the simulation result. According to the experimental results, the selector should be higher than 1 turn.

The increase in *H*_s_ exhibited a turning point at 14 mm as the *h*_s_ increased. Consequently, a high *h*_s_ value would consume a high amount of material and increase the solidification time. The maximum *h*_s_ was 14 mm under these study conditions and the maximum *h*_s_ was recommended to be 1.17 *d*_s_, according to the proportional relationship between *h*_s_ and *d*_s_ (Equation (11)). Additionally, the upper and lower spiral tunnels could not coincide with each other in the casting shell, which meant that the *h*_s_ should be at least double the shell thickness *h*_m_; also, Equation (12) was considered to fully include the role of Effect 2. Therefore, the minimum value of *h*_s_ could be calculated as follows:

(14) $${\left(h_{s}\right)}_{\min}=\left\{ \begin{array}{l} 2h_{m},\;\;\;2h_{m}>d_{w}\tan(\theta )\\ d_{w}\tan(\theta ),\;d_{w}\tan(\theta )>2h_{m} \end{array}\right.$$

#### 4.1.4. Effects of *d*_s_

**Figure 15:**
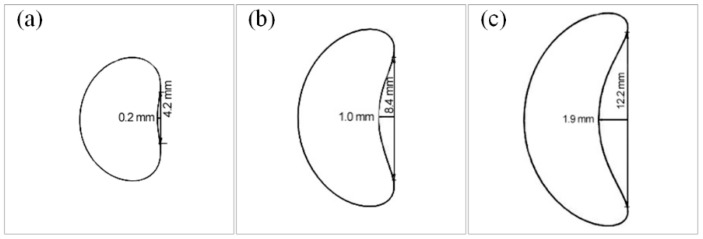
Cross-section shapes of spiral part with different *d*_s_ (*h*_s_ = 14 mm and *d*_w_ = 5 mm): (**a**) *d*_s_ = 8 mm, *θ* = 29.1° and bending degree (BD) = 0.12; (**b**) *d*_s_ = 10 mm, *θ* = 24.0° and BD = 0.24; and (**c**) *d*_s_ = 14 mm, *θ* = 17.6° and BD = 0.31.

According to Equation (11), the *d*_s_ is inversely proportional to tan*θ* when the *h*_s_ is constant. Figure 15 presents the cross sections of spiral parts with different *d*_s_. In this figure, the *d*_s_ increased from 8 mm to 14 mm, the BD increased from 0.12 to 0.31 and the *θ* decreased from 29.1° to 17.5°. The Effect 1 was enhanced due to the increase of BD, which led to the grain enhancement. Additionally, the decrease of *θ* enhanced the effect of Effect 2 and the grain selection effect was further enhanced. Therefore, the grain selection was significantly enhanced as the *d*_s_ increased.

**Figure 16:**
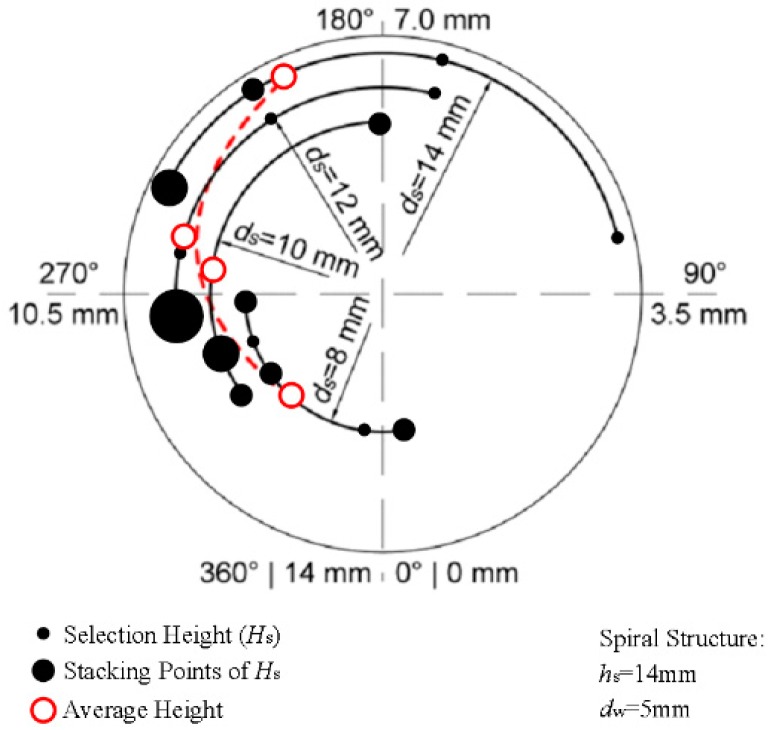
Comparison of *H*_s_ in selectors with different *d*_s_ during simulation (10 trials) with *h*_s_ = 14 mm and *d*_w_ = 5 mm.

Figure 16 presents the simulation results of *H*_s_ in selectors with different *d*_s_. In this figure, the radius of the arc was equal to *d*_s_ /2, the arc degree corresponded to the *H*_s_ and the arc range indicated the range in which *H*_s_ might occur. The average height of *H*_s_ apparently decreased and the grain selection effect was enhanced as the *d*_s_ increased, which matched the aforementioned theory analysis. However, the appearance range of *H*_s_ widened and the randomness of the grain selection was enhanced. In addition, the crystal selection height was higher than the corresponding pitch value *h*_s_ when the *d*_s_ was small—therefore, the selector must be higher than 1-turn to ensure the successful selection of a single crystal structure.

**Figure 17:**
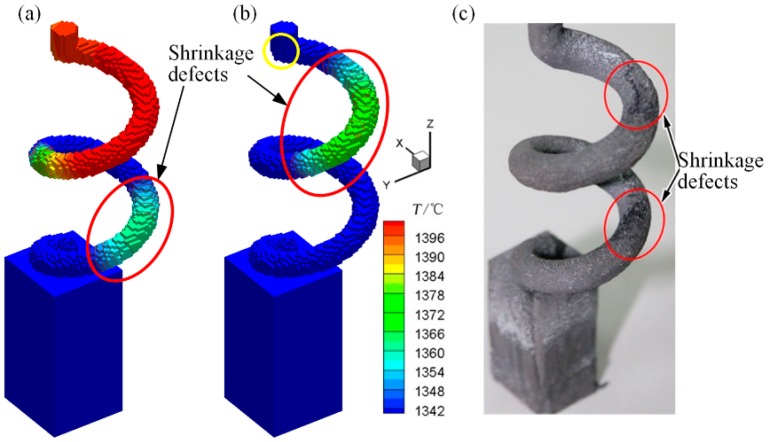
Simulation analysis and experiment of shrinkage defects in selector with *h*_s_ = 14 mm and *d*_s_ = 14 mm: (**a**) temperature field (*t* = 20 min); (**b**) temperature field (*t* = 23 min) and (**c**) experimental result.

The aforementioned analysis proved that an increase in *d*_s_ could enhance the selection effect, whereas excessively high *d*_s_ could create a loose crystal structure, an imbalanced isothermal surface, and shrinkage defects in practical applications, as presented in Figure 17. In this figure, the loose structure changed the visual radiation angle when the *d*_s_ value was high (14 mm), resulting in the priority cooling of certain local sections (red areas in Figure 17a,b) of the DS casting. These liquid regions completed the corresponding solidification and might have formed shrinkage defects, as presented in Figure 17c. Additionally, the liquid region at the top of the spiral part (yellow areas in Figure 17b) did not follow this solidification sequence. Stray grains might have nucleated and grown in this undercooled region, which would directly increase the failure risk of the grain selection process.

The structural shape and DS process should be considered in the design of *d*_s_. Under the authors’ study conditions, the upper limit of *d*_s_ was 14 mm. Concerning Equation (11), the minimum *d*_s_ should be as follows:

(15) $${\left(d_{s}\right)}_{\min}=\left\{ \begin{array}{l} 2h_{m}\arctan(\theta )/\pi ,\;2h_{m}\arctan(\theta )/\pi \;>d_{w}/\pi \\ d_{w}/\pi ,\;\;\;\;\;d_{w}/\pi \;>2h_{m}\arctan(\theta )/\pi \end{array}\right.$$

### 4.2. Improvement of Spiral Structure

The structures of spiral selectors that are used in engineering are quite different. Stray grains or bi-crystal defects in a single crystal blade product usually appear as irrational structures within the spiral selector.

**Figure 18:**
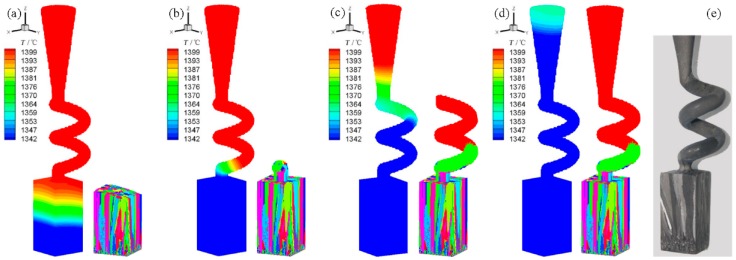
Mushy zone distribution, microstructure simulation results and experiment validation of improved spiral selector (*h*_s_ = 14 mm and *d*_s_ = 12 mm): (**a**) *t* = 10 min; (**b**) *t* = 15 min; (**c**) *t* = 20 min; (**d**) *t* = 25 min and (**e**) experimental result.

An improved spiral selector structure was proposed based on the spiral selection mechanism and the parameter design criteria, with the parameters of *h*_s_ = 14 mm, *d*_s_ = 12 mm, *d*_w_ = 5.0 mm, and *h*_b_ = 30 mm. A 2-turn spiral was designed to ensure the successful selection of a single crystal structure. Figure 18 presents the simulation and experimental results of this improved selector during DS. During the early solidification stage, the mushy zone appeared slightly convex and inclined due to the eccentric arrangement of the crystal. In contrast, this inclination degree was low and subsequently barely affected the growth and orientation of the grains. As the DS continued, the mushy zone completed the sequential solidification process and quickly passed through the spiral part, until it reached the top of the spiral selector. Finally, a single crystal was successfully selected. The appearance height was 9.1 mm in the simulations compared to 8.4 mm in the experiments and the orientation deflection angle was 3° in the simulations compared to 7° in the experiments. These simulation differences were mainly caused by the randomness of the grain selection process. This improved spiral selector could accurately and efficiently select the single crystal structures in both the experiments and simulations.

## 5. Conclusions

Different spiral selectors were studied through experimental and simulation methods with the 3D printing technology. The grain selection mechanism was analyzed and verified, the criteria for the selector parameters were proposed and the spiral structure was improved. The main conclusions were as follows:

(1) Thermal transfer and microstructure models based on CA-FD were built to simulate the temperature variation and microstructure evolution in the spiral selector during the single crystal superalloy casting.
(2) Temperature curves and cooling rates in different points of the selector were compared through experimentation and simulations. The corresponding average errors were below 1.5%. The microstructure evolutions in the selector were investigated through EBSD and simulations and agreed well. The grain selection mechanism was further analyzed and verified with simulations. The design rules of the selector parameters, based on the two selection effects, were proposed.
(3) The effects of the spiral structure parameters on the grain selection were studied through precise casting of different spiral selectors fabricated with 3D printing. The design criteria for these parameters, such as the starter block height (*h*_b_), the spiral tunnel diameter (*d*_w_), the spiral pitch (*h*_s_) and the spiral diameter (*d*_s_), were proposed, whereas the spiral selector structure was improved. The results demonstrated that this improved selector could accurately and efficiently select a single crystal.

## Figures and Tables

**Table 1:** Structure parameters of the spiral grain selectors in the experiment.

ES1 Group (*d*_s_ = 12 mm, *d*_w_ = 5 mm)				ES2 Group (*h*_s_ = 14 mm, θ = 30°)
*h*_s_/mm	θ/°	*h*_s_/mm	θ/°	*d*_w_/mm
8.7	13.0	16.8	24.0	3
11.2	16.5	20.1	28.0	5
14.0	20.2	27.4	37.0	7

**Table 2:** Physical parameters in the simulation.

Parameters	Values	Parameters	Values
Liquidus/K	1672	Thermal conductivity of alloy/J·(m·s·K)^−1^	33.2
Solidus/K	1615	Specific heat of alloy/kJ·(kg·K) ^−1^	0.773
Density of alloy/kg·m^−3^	8780	Liquidus slope/°C·%^−1^	−3.95
Latent heat/kJ·kg^−1^	99	diffusion coefficient in liquid(*D*_L_)/m^2^·s^−1^	3.6 × 10^−9^
Concentration/wt %	39.006	diffusion coefficient in liquid (*D*_S_)/m^2^·s^−1^	1.0 × 10^−12^
Density of shell/kg·m^−3^	2500	Thermal conductivity of shell/J·(m·s·K)^−1^	1.95
Specific heat of shell/kJ·(kg·K)^−1^	1.323	Cast-shell interface resistance/m^2^·K·W^−1^	1.8 × 10^−2^
